# Use of Covered Stents in Cannulation Sites as a Last Option to Salvage Failing Vascular Access

**DOI:** 10.1177/15266028221116745

**Published:** 2022-08-16

**Authors:** Johannes W. Drouven, Cor de Bruin, Arie M. van Roon, PhD, Akin Ozyilmaz, Job Oldenziel, Reinoud P. H. Bokkers, Clark J. Zeebregts

**Affiliations:** 1 Departments of Surgery, Division of Vascular Surgery, University Medical Center Groningen, University of Groningen, Groningen, The Netherlands; 2 Internal Medicine, Division of Vascular Medicine, University Medical Center Groningen, University of Groningen, Groningen, The Netherlands; 3 Internal Medicine, Division of Nephrology, University Medical Center Groningen, University of Groningen, Groningen, The Netherlands; 4 Radiology, Medical Imaging Center, University Medical Center Groningen, University of Groningen, Groningen, The Netherlands

**Keywords:** vascular access, covered stent graft, puncture, hemodialysis, endovascular treatment/therapy

## Abstract

**Purpose:**

Controversy exists regarding the treatment of recurrent stenosis in vascular access at cannulation sites with a covered stent as repeated cannulation may damage the stent. The purpose of this study was to review covered stent placement at cannulation sites to salvage failing vascular access.

**Materials and methods:**

A total of 11 patients were included for the purpose of this study. Eight patients (72.7%) received a covered stent due to recurrent stenosis, 2 (18.2%) due to an acute occlusion, and in 1 case (9.1%), the covered stent was used to repair a damaged polytetrafluoroethylene arteriovenous graft (PTFE AVG).

**Results:**

Primary patency after stent placement was 40.9% at 6 months, primary-assisted patency was 79.5% at 12 months, and secondary patency was 80% at 24 months. No significant problems were observed during the dialysis sessions after stent placement. The intervention rate per patient-year was not significantly different before or after covered stent placement, at 3.8 (IQR=9.5) interventions per year versus 2.5 (IQR=3.0) interventions per year (p=0.280).

**Conclusion:**

In conclusion, treating failing vascular access with problems at cannulation sites with covered stents can be considered.

**Clinical Impact:**

Treating vascular access stenosis at cannulation sites with covered stents can successfully prolong vascular access life.

## Introduction

Long-term vascular access function and patency are essential to the survival of hemodialysis patients.^1,2^ Late vascular access complications, such as aneurysm formation at the puncture site, recurrent stenosis, or thrombosis are commonly seen, with a higher incidence in AVGs than in arteriovenous fistulae (AVFs).^2^

In recent years, use of covered stents has become more common to treat recurrent stenoses in the venous outflow or anastomotic tract of the vascular access.^3,4^ Covered stents can prolong vascular access life and have shown improved patency in failing grafts.^5^ Ultimately, covered stents might be used in failing AVFs to prevent access abandonment.^6^ The treatment of recurrent stenosis at cannulation sites with a covered stent remains controversial as repeated cannulation may damage the fabric or struts.^7–9^ Off-label use of covered stents at cannulation sites has not been described extensively and is mainly reported for patients with vascular access-related pseudoaneurysms.^10,11^

The purpose of this study was to retrospectively review covered stent placement at cannulation sites to salvage failing vascular access. We aimed to determine effectiveness and durability of repeated cannulation of covered stents for hemodialysis.

## Materials and Methods

### Study Design

This is a single-center retrospective cohort study. Data of patients with chronic renal dysfunction requiring hemodialysis treated in our single center were analyzed. Data were obtained from a prospectively maintained database of patients treated between November 2004 and December 2021. A total of 856 patients were included in this database.

The institutional review board approved the study (METc 2018/015). Research studies involving the retrospective review, collection, and analysis of patient records do not fall under the Dutch Medical Research Involving *Human Subjects Act* (WMO) and therefore individual patient informed consent was not required. The opt out registry of the institution was consulted to find out whether patients had objected to participating in scientific research. Storage and analysis of data were anonymized.

Patient demographics and characteristics, comorbidity, type of vascular access, procedural details, and postoperative outcomes were obtained from the database. All patient-related data were pseudoanonymized prior to analyses.

### Indications for Interventions

Vascular access stenosis was defined as the presence of a peak systolic velocity greater than 375 cm/sec or with a vessel diameter smaller than 2.0 mm.^12^ Patients with suspected vascular access thrombosis were referred to the emergency department or the dialysis clinic to confirm the diagnosis. Thrombosis was defined as a lack of thrill or pulse of the vascular access, confirmed by an absence of flow on duplex ultrasound examination. Covered stent placement in vascular access cannulation zones was only considered in patients with no other options than vascular access abandonment, due to recurrent thrombosis, stenosis, or other problems.

### Covered Stent Placement

All procedures were performed under local anesthesia. Standard antibiotic prophylaxis was given prior to the procedure. The Seldinger technique was used to obtain access under ultrasound guidance. The puncture site was determined by the localization of the stenosis or thrombus and the type of vascular access. In patients with an AVG, the AVG itself was cannulated. In patients with an AVF, the vein was cannulated. For cases of recurrent stenosis, a standard angioplasty technique was used with noncompliant balloons. For cases of recurrent thrombosis, an emergency thrombectomy procedure was performed as described earlier.^13^ After these procedures, vascular access angiography was performed to determine the length of the lesion and the diameter of the target vessel. All covered stents used in this study were GORE VIABAHN (W. L. Gore and Associates, Flagstaff, AZ, USA). The covered stent diameter used was usually 1 mm greater than the diameter of the target vessel. Covered stents were inserted through an 8F sheath over a 0.035 inch hydrophilic guidewire and deployed at the intended treatment zone. Post-deployment balloon dilatation of the covered stent was performed with a noncompliant balloon matching the diameter of the covered stent. Completion angiography was performed and remaining significant stenotic segments (>50%) were treated with balloon angioplasty. External manual compression was performed to obtain hemostasis; occasionally, an Angio-seal 8F closure device was used to obtain hemostasis. Antiplatelet therapy was started after the procedure and standard dose acetylsalicylic acid was administered.

### Follow-up

All patients were briefly monitored in the post-procedural period and were generally discharged the same day. Cannulation of the covered stent was allowed at the following dialysis session and was performed or supervised by a single experienced dialysis nurse. All stent grafts were cannulated with ultrasound guidance. Reinterventions, complications, and other events were recorded. Primary, primary-assisted, and secondary patency were defined as described earlier.^2^

After discharge, routine physical examination and ultrasound dilution flow measurements were performed with either a Transonic HD01 plus Hemodialysis Monitor (Transonic Systems Inc., Ithaca, NY, USA) or a Fresenius 5008S CorDiax dialysis machine (Fresenius Medical Care, Bad Homburg, Germany).

### Statistical Analysis

Data are presented as medians including IQR or numbers including percentages. The Wilcoxon signed rank test was used to calculate differences between interventions before and after stent placement. Kaplan–Meier survival analysis and the life table method were used to calculate patency rates. SPSS Version 24 (SPSS Inc., Chicago, IL, USA) was used for analysis.

## Results

A total of 11 patients who underwent covered stent placement at cannulation zones for salvaging failing vascular access between 2018 and 2021 were included in our study. Table 1 details the characteristics of the included patients. Median age at stent placement was 63.0 years (IQR=19.0). Four procedures were performed in patients with a brachiocephalic AVF, 5 in patients with an AVG, and 2 in patients with a basilic vein transposition. The median age of the vascular access was 3.2 years at the time of covered stent placement and the median number of interventions per year was 3.8 at the time of covered stent placement. Eight patients were treated for recurrent stenosis, 2 for an acute occlusion of the vascular access, and 1 for a failing polytetrafluoroethylene arteriovenous graft (PTFE AVG) due to repeated cannulation. In the last patient, the indication for the covered stent placement was PTFE disintegration due to repeated cannulation.

**Table 1.** Patient Characteristics.

Number of cases	11
Age at stent placement (years)	63.0 (19.1)
Sex	
Male	4 (36.4)
Female	7 (63.6)
BMI (kg/m^2^)	28.7 (7.6)
Diabetes mellitus	3 (27.3%)
Access type	
Brachiocephalic AVF	4 (36.4%)
AVG	5 (45.4%)
Basilic vein transposition	2 (18.2%)
Indication for covered stent	
Recurrent stenosis	8 (72.7%)
Occlusion	2 (18.2%)
Failing PTFE AVG	1 (9.1%)
Age of vascular access (years)	3.2 (4.8)
Number of interventions per year	3.8 (9.5)

Data are presented as numbers with percentages or medians including IQR.

Abbreviations: AVF, arteriovenous fistula; AVG, arteriovenous graft; BMI, body mass index; PTFE, polytetrafluoroethylene.

### Stent Placement and Cannulation

Ten patients were treated with a 7 mm diameter covered stent and the remaining case was treated with an 8 mm diameter stent. Data on stent placement, type of vascular access, and location of the treated segment are shown in Table 2. Technical success was 100%. Additional balloon angioplasty of remaining access stenosis was performed in 9 cases (81.2%). Table 3 shows the long-term outcomes. The median time to cannulation of vascular access through the covered stent was 13 days (IQR=34 days). In the patient with a failing PTFE AVG, cannulation of the covered stent was delayed to minimize the risk of false aneurysm formation or persistent bleeding. All 11 patients were on 2-needle dialysis, 5 with both needles through the covered stent (45.5%) and the remaining 6 with only 1 needle through the covered stent (54.5%).

**Table 2.** Covered Stent Data.

	Type of vascular access	Covered stent size and length	Location of the treated segment
Case 1	PTFE forearm loop	7×50 mm and 7×100 mm	Stenosis in loop and venous anastomosis
Case 2	PTFE straight	7×50 mm and 7×100 mm	Multiple stenosis in PTFE
Case 3	Brachiocephalic AVF	7×50 mm, 7×50 mm and 7×100 mm	Multiple stenosis in venous outflow tract
Case 4	PTFE straight	7×50 mm and 7×100	Stenosis in PTFE
Case 5	Basilic vein transposition	7×100 mm	Stenosis at venous puncture site
Case 6	PTFE forearm loop	7×50 mm and 7×100 mm	Multiple stenosis in PTFE and venous anastomosis
Case 7	Basilic vein transposition	7×50 mm and 7×100 mm	Stenosis at venous puncture site
Case 8	Brachiocephalic AVF	8×50 mm and 8×50 mm	Stenosis at venous puncture site and cephalic arch
Case 9	PTFE straight	7×50 mm	Stenosis in PTFE
Case 10	Brachiocephalic AVF	7×150 mm	Stenosis at venous puncture site
Case 11	Brachiocephalic AVF	7×100 mm	Senosis at venous puncture site

Abbreviations: AVF, arteriovenous fistula; PTFE, polytetrafluoroethylene.

**Table 3.** Outcomes.

Number of cases	11
Follow-up time (months)	
Before stent placement	39.1 (78.1)
After stent placement	19.0 (34.0)
Days to cannulation	13 (34)
Two-needle hemodialysis	5 (45.5%)
Hemodialysis problems	0 (0%)
Number of interventions	
Before covered stent	7 (11)
After covered stent	3 (6)
Intervention rate (interventions per year)	
Before covered stent	3.8 (9.5)
After covered stent	2.5 (3.0)

Data are presented as numbers with percentages or medians including IQR.

### Long-term Follow-up

**Figure 1.**
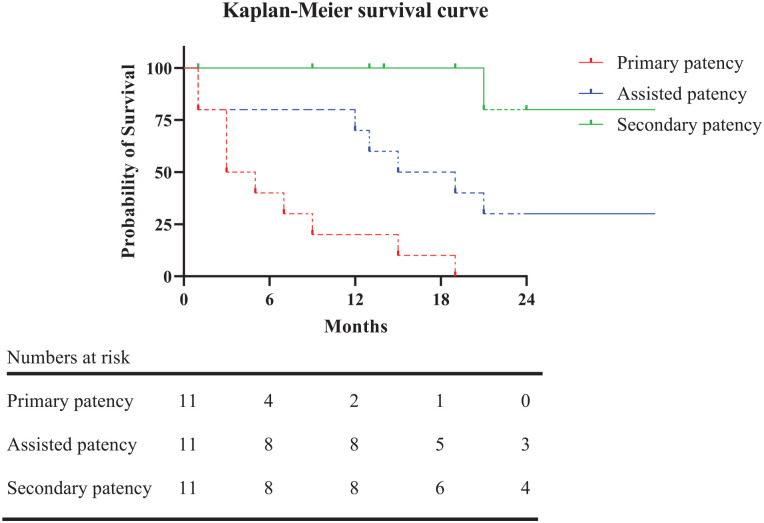
Kaplan–Meier survival curve. Dashed lines represent a value with a standard error of more than 10%.

Median follow-up time from vascular access creation to stent placement was 39.1 months (78.1). The median follow-up time was 19.0 months after stent placement (IQR=24.0). Primary patency after stent placement was 40.9% at 6 months (95% confidence interval [CI] [0.32–0.50]), primary-assisted patency was 79.5% at 12 months (95% CI [0.72–0.87]), and secondary patency 80.0% at 24 months (95% CI [0.69–0.91]). Due to the small number of patients and events, a 6 month interval was chosen for primary patency, a 12 month interval for primary-assisted patency, and a 24 month interval for secondary patency. Corresponding Kaplan–Meier survival curves are shown in Figure 1. The intervention rate per patient-year was not significantly different before or after covered stent placement, at 3.8 (IQR=9.5) interventions per year versus 2.5 (IQR=3.0) interventions per year (p=0.280). In one patient, the vascular access was removed after 21 months due to secondary AVG infection—an infected hematoma following an endovascular procedure. Another patient did not respond to antiplatelet treatment after covered stent placement, following recurrent occlusions and a surgical revision. After switching to different antiplatelet agents, no additional surgical revisions were required.

**Figure 2.**
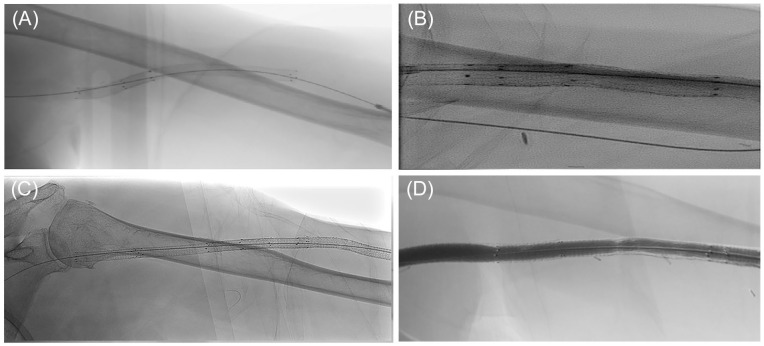
X-ray of covered stents in different patients. (A) After 6 months of cannulation. (B) After approximately 12 months of cannulation. (C) After 18 months of cannulation. (D) An angiographic example of a covered stent after approximately 2 years of cannulation.

None of the cases required surgical revision for stenosis or abandonment after covered stent placement. All reinterventions included endovascular procedures for recurrent stenosis, outside of the previous treated segment with the covered stent. In addition, no edge stenosis or recurrent stenosis in the previously treated segment was observed. No other significant problems were observed during the dialysis sessions after stent placement. Figure 2 shows X-rays of different patients after 6, 12, and 18 months of cannulation. No significant changes to the integrity of the covered stent can be observed.

## Discussion

In this study, we showed that failing vascular access due to recurrent stenosis in cannulation zones can be successfully salvaged with covered stents, after which cannulation is still possible. Promising patency rates were observed with a median follow-up time of 19 months. Despite repeated cannulation of the covered stent, similar intervention rates were observed before and after stent placement. During hemodialysis, there were no problems with needle access and none of the patients suffered from flow-limiting stent fractures. In one case, the AVG and covered stent were removed after 21 months due to infection of the AVG. Whether the covered stent was related to the infection was unclear, but infections are frequently seen in patients with an AVG.^1^

Covered stent placement in dysfunctional vascular access can be considered as an effective treatment and might help preserve function and prevent access abandonment.^6^ The use of covered stents in cannulation zones remains controversial, however. Covered stents, such as the GORE VIABAHN (W.L. Gore and Associates, Flagstaff, AZ, USA) used in our study, are CE-marked for the treatment of stenosis in the dialysis access circuit. The instructions for use however state that cannulation may result in damage to the fabric or nitinol stent. Previous studies comparing the use of covered stents for failing vascular access with conventional angioplasty therefore considered the positioning of covered stents in cannulation zones as an exclusion criterion.^9^

Experience with repeated covered stent cannulation for hemodialysis remains limited. Several studies assessed the use of covered stents in AVG pseudoaneurysm repair, following repeated cannulation for hemodialysis of the covered stent.^9–11^ Vesely^9^ published one of the first series of cases in 2005 in which a covered stent was used to repair hemodialysis graft–related pseudoaneurysms. They reviewed 11 cases and found that the use of a covered stent for exclusion of AVG pseudoaneurysms can be considered as an effective treatment. Immediate cannulation of the covered stents was allowed initially, but due to leaks at the covered stent cannulation sites they advocated a 30-day no-cannulation policy at the pseudoaneurysm site.

Rhodes and Silas^11^ evaluated the effect of routine venipuncture for hemodialysis on the durability of covered stents in 6 patients who were treated for AVG pseudoaneurysms. In their series, they showed that covered stents (Wallgraft, Boston Scientific, Galway, Ireland) could withstand the routine cannulation required for hemodialysis without flow-limiting distortion of the stent. In 2016, Wong et al^10^ published a study of 35 patients, wherein 37 covered stents were used to repair AVG pseudoaneurysms. Cannulation of the covered stent was allowed for hemodialysis in 28 patients. However, data on cannulation timing and numbers of the stent grafts are missing.

Ours was a retrospective study that reviewed the results of 11 cases. The major weaknesses are therefore the limited sample size and the retrospective nature of the study. Literature on the treatment of cannulation zone problems in vascular access with covered stents remains scarce.

Based on our results, treating vascular access stenosis at cannulation sites with covered stents should be considered to prolong vascular access life with similar intervention rates and acceptable patency rates in these fragile hemodialysis patients.
